# Melatonin Regulatory Mechanisms and Phylogenetic Analyses of Melatonin Biosynthesis Related Genes Extracted from Peanut under Salinity Stress

**DOI:** 10.3390/plants9070854

**Published:** 2020-07-06

**Authors:** Abdelaleim I. ElSayed, Moncef Boulila, Mohammed S. Rafudeen, Azza H. Mohamed, Sonali Sengupta, Mostafa Rady, Ahmad A. Omar

**Affiliations:** 1 Biochemistry Department, Faculty of Agriculture, Zagazig University, Zagazig 44519, Egypt; aelsayed@zu.edu.eg; 2 Université de Sfax- Institut de l’Olivier- B.P. 14, Ibn Khaldoun, Sousse 4061, Tunisia; boulila.moncef@yahoo.fr; 3 Department of Molecular and Cell Biology, University of Cape Town, Private Bag, Rondebosch 7701, South Africa; Suhail.Rafudeen@uct.ac.za; 4 Agricultural Chemistry Department, Faculty of Agriculture, Mansoura University, Mansoura 35516, Egypt; 5 Citrus Research and Education Center, University of Florida, IFAS, Lake Alfred, FL 33850, USA; 6 School of Plant, Environmental and Soil Sciences, Louisiana State University, Agricultural Center, Baton Rouge, LA 70808, USA; SSengupta@agcenter.isu.edu; 7 Botany Department, Faculty of Agriculture, Fayoum University, Fayoum 63514, Egypt; mmr02@fayoum.edu.eg

**Keywords:** antioxidant defense, *Arachis hypogaea*, melatonin, phylogenetic analysis, salinity stress, gene expression, *ASMT*, *TDC*, *T5H*

## Abstract

Melatonin improves the tolerance of plants to various environmental stresses by protecting plant cells against oxidative stress damage. The objective of the current study was to determine whether exogenous melatonin (MT) treatments could help protecting peanut (*Arachis hypogaea*) seedlings against salinity stress. This was achieved by investigating enzymatic and non-enzymatic antioxidant systems and the expression of melatonin biosynthesis related genes in response to salinity stress with or without exogenous MT. The results showed a significant increase in the concentrations of reactive oxygen species (ROS) in peanut seedlings under salinity stress. The exogenous application of melatonin decreased the levels of ROS through the activation of antioxidant enzymes in peanut seedlings under salinity stress. Transcription levels of melatonin biosynthesis related genes such as N-acetylserotonin methyltransferase (*ASMT1*, *ASMT2*, *ASMT3*), tryptophan decarboxylase (*TDC*), and tryptamine 5-hydroxylase (*T5H*) were up-regulated with a 150 µM melatonin treatment under salinity stress. The results indicated that melatonin regulated the redox homeostasis by its ability to induce either enzymatic or non-enzymatic antioxidant systems. In addition, phylogenetic analysis of melatonin biosynthesis genes (*ASMT1*, *ASMT2*, *ASMT3*, *TDC*, *T5H*) were performed on a total of 56 sequences belonging to various plant species including five new sequences extracted from *Arachis hypogaea* (*A. hypogaea*). This was based on pairwise comparison among aligned nucleotides and predicted amino acids as well as on substitution rates, and phylogenetic inference. The analyzed sequences were heterogeneous and the *A. hypogaea* accessions were primarily closest to those of *Manihot esculenta*, but this needs further clarification.

## 1. Introduction

Globally, salinity stress is a major challenge to the agricultural sector impacting food security. Salinity stress results in harmful impacts on plant cells by either water shortfall triggered by osmotic stress or the effect of excess sodium ions on key biochemical processes [1]. To limit these deleterious effects, plants utilize several biochemical and molecular strategies, such as synthesis of compatible osmolytes, induction of antioxidative enzymes, modification to the photosynthetic pathway, alterations in the membrane structure, and regulation of gene expression [2]. Furthermore, salinity stress leads to the production of reactive oxygen species (ROS). The harmful effect of ROS includes DNA alteration, lipids oxidation, inhibition of amino acid metabolism, and interrupts the activities of several enzymes, in addition to the membrane’s damage [3]. In order to alleviate the harmful effects of reactive oxidative stress, plants detoxify ROS either by activation of non-enzymatic antioxidants, such as carotenoids, glutathione (GSH), and ascorbic acid (AsA), or through the up-regulating of antioxidative enzymes including superoxide dismutase (SOD), catalase (CAT), ascorbate peroxidase (APX), glutathione reductase (GR), peroxiredoxins (Prxs), and glutathione peroxidase (GPX) [4].

Melatonin (N-acetyl-5-methoxytryptamine), a low-molecular-weight molecule, is widespread in evolutionarily distant organisms and exhibits numerous biological functions in species ranging from bacteria to mammals [5]. In plants, it has been implicated as a growth regulator and protecting plants against various adverse environmental conditions [6,7,8,9,10]. Although several reports demonstrated the role of melatonin in alleviating the adverse impacts of biotic or abiotic stresses in plants, the functional and regulatory roles of melatonin are not yet fully understood. Melatonin is synthesized in the plant cells from the amino acid tryptophan. This process involves four different enzymes; N-acetylserotonin methyltransferase (*ASMT*), tryptophan decarboxylase (*TDC*), tryptamine 5-hydroxylase (*T5H*), and serotonin N-acetyltransferase (*SNAT*) [11,12]. These melatonin biosynthetic genes have been cloned and characterized in rice [11,12] but the regulation of melatonin synthesis in plants remains unclear. In plants, melatonin plays pivotal roles in stress mitigation through the enhancement of the photosynthesis capacity, development of cellular redox homeostasis, mitigation of oxidative stress, and regulating the expression of stress reactive genes, therefore, modulate signal transduction factors [13,14]. Ke et al. [15] stated that melatonin is engaged in different responses to environmental factors in plants. Moreover, melatonin mitigates salt stress by regulating polyamine metabolism in wheat seedlings. In cucumber seedlings grown under salt stress conditions, applying melatonin increased antioxidant enzyme activity, improved cell viability, and reduced malondialdehyde (MDA) content [16]. It has been shown that melatonin activates antioxidant systems, including the ascorbate–glutathione cycle and increased the concentration of ascorbic acid and glutathione [14,17]. Moreover, several reports demonstrated that salinity stress can enhance the melatonin level in plant roots [18,19]. Chen et al. [20] reported that exogenous melatonin improved seed germination in cotton, enhancing osmotic substances and adjusting ion homeostasis under salt-stress. Further results proved that there is a correlation between melatonin level and cotton seed germination. Cen et al. [21] also stated that an exogenous application of melatonin improved alfalfa seed germination and reduced oxidative damage under salinity stress. A previous study revealed that an exogenous treatment of melatonin alleviated the adverse effects of salinity stress in *Malus hupehensis*, *Citrullus lanatus*, and *Helianthus annuus* [13,19,22]. Nevertheless, it remains uncertain whether such effect of melatonin against salinity stress is a common phenomenon of other plant species. Moreover, the mechanism of melatonin-mediated salinity stresses tolerance in plants is still not understood completely.

Peanut (*Arachis hypogaea* L.) is one of the essential oilseeds and food legume crops. However, high salinity in the soil is a major abiotic stress controlling peanut yield and productivity [23]. Little is known regarding the relative levels of salinity tolerance of peanut although it has been reported that salinity adversely affects peanut germination, growth, and seed quality. The current study aimed to investigate the regulatory mechanism controlling melatonin-mediated salinity tolerance in peanut in terms of enzymatic and non-enzymatic antioxidant systems. Moreover, the transcriptional response of genes encoding melatonin biosynthesis was analyzed. This study provides an insight into the functional and regulatory mechanism of melatonin in peanut under salinity stress. Furthermore, the relationship between melatonin biosynthesis related genes and salt tolerance of peanut plant was analyzed. Additionally, the current study focused on the phylogenetic relationships of four newly sequenced melatonin biosynthesis encoding genes amplified from *A. hypogaea* (ASMT1, 2, and 3, and T5H) as well as from other plant species whose sequences were retrieved from GenBank.

## 2. Results

### 2.1. Levels of Malondialdehyde (MDA), Hydrogen Peroxide (H_2_O_2_), and Proline under Salinity Stress and Melatonin (MT) Treatments

**Figure 1:**
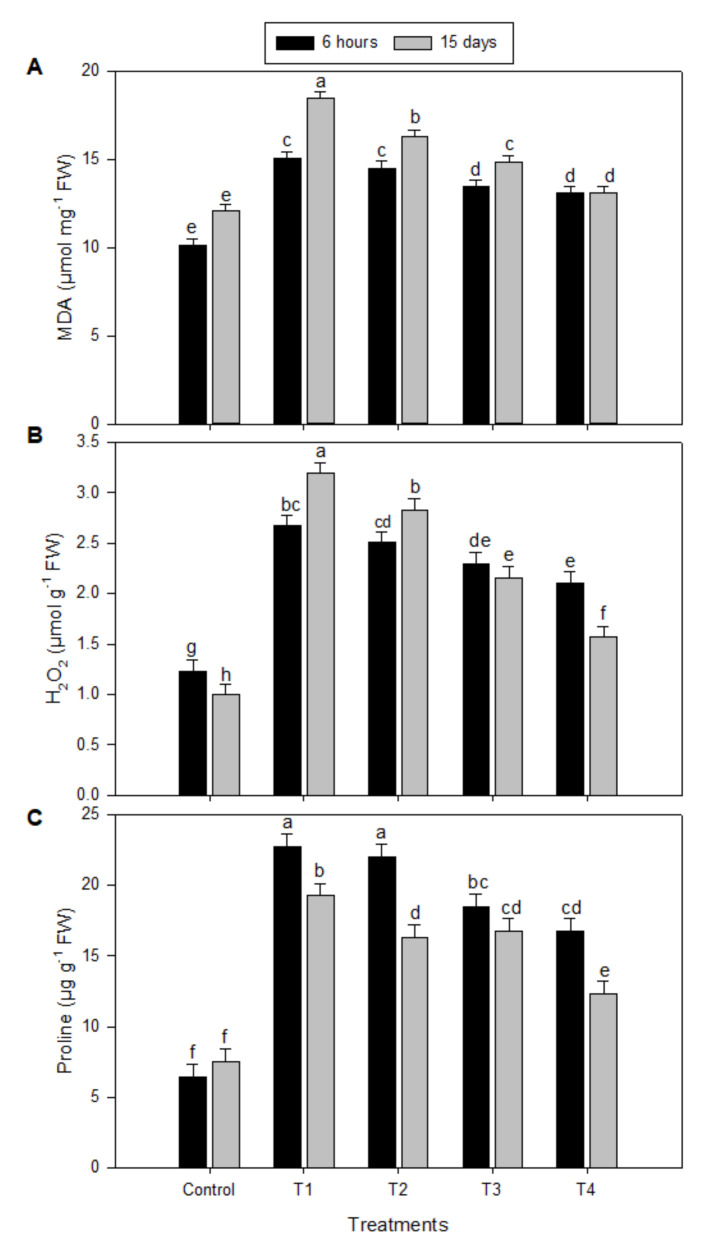
Effect of exogenous melatonin on malondialdehyde (MDA), H_2_O_2_, and proline contents (µmol g^−1^ FW) in peanut seedlings under salinity stress after 6 h and 15 days. (**A**) Malondialdehyde (MDA), (**B**) H_2_O_2_, and (**C**) proline. Data are means ± SD (*n* = 3) from three independent experiments. The bars labelled with different letters are significantly different at *p* ≤ 0.05. The significant difference marked by different letters was calculated using the Student’s *t*-test and further analyzed with the Fisher LSD test, at *p* ≤ 0.05. The legends are as follows: Control: 0 NaCl + 0 melatonin (MT), T1: 150 mM NaCl + 0 MT, T2: 150 mM NaCl + 50 μM MT, T3: 150 mM NaCl + 100 μM MT, T4: 150 mM NaCl + 150 μM MT.

The levels of MDA (measured to indicate the level of lipid peroxidation) (Figure 1A) and H_2_O_2_ (Figure 1B) were increased significantly with a significant increase in the proline level (Figure 1C) in the leaves of peanut seedlings as a result of treatment of salinity stress (T1) compared to control (Figure 1). However, the exogenous use of 100 µM (T3) or 150 µM (T4) of MT for salt-treated seedlings resulted in significant reductions in the levels of MDA, H_2_O_2_, and proline, but control values had not been reached (Figure 1A–C, respectively). The exogenous use of 50 µM of MT (T2) for salt-treated seedlings resulted in an unconvincing decrease compared to 100 or 150 µM of MT (T3 and T4, respectively). Although elevations in the levels of MDA and H_2_O_2_ were explored in 15-day-stressed seedlings (with some fluctuations), proline level behaved a reverse trend compared to 6-hour-stressed seedlings.

### 2.2. Levels of Antioxidant System Components in Peanut Seedlings under Salinity Stress and Melatonin (MT) Treatments

**Figure 2:**
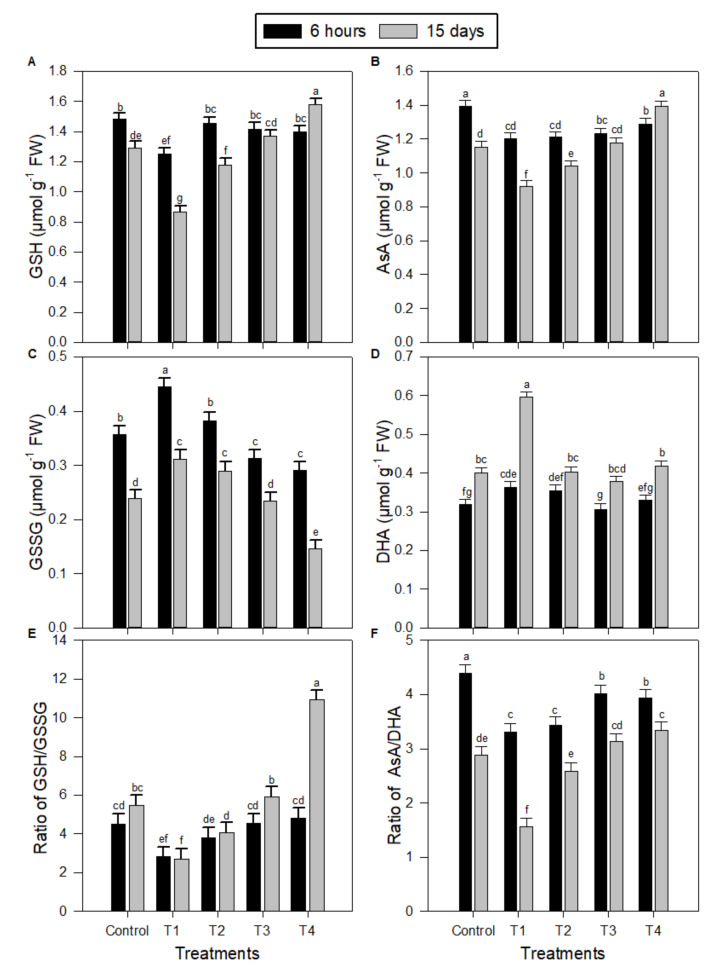
Effect of salinity stress alone or combined with exogenous melatonin on antioxidant system components in peanut seedlings after 6 h and 15 days. (**A**) Reduced glutathione (GSH), (**B**) ascorbate (AsA), (**C**) oxidized glutathione (GSSG), (**D**) dehydroascorbate (DHA), (**E**) the ration of GSh/GSSH, and (**F**) the ratio of AsA/DHA. Data are means ± SD (*n* = 3) from three independent experiments. The bars labelled with different letters are significantly different. The significant difference marked by different letters was calculated using the Student’s *t*-test and further analyzed with the Fisher LSD test, at *p* ≤ 0.05. The legends are as follows: Control: 0 NaCl + 0 MT, T1: 150 mM NaCl + 0 MT, T2: 150 mM NaCl + 50 μM MT, T3: 150 mM NaCl + 100 μM MT, T4: 150 mM NaCl + 150 μM MT.

The contents of reduced glutathione (GSH) (Figure 2A), ascorbate (AsA) (Figure 2B), the ratio of GSH/oxidized glutathione (GSSG) (Figure 2E), and the ratio of AsA/dehydroascorbate (DHA) (Figure 2F) were significantly decreased, while the contents of GSSG (Figure 2C) and DHA (Figure 2D) were significantly increased in the leaves of peanut seedlings as a result of treatment of salinity stress (T1) compared to control (Figure 2). However, the exogenous use of MT applied at 50–150 µM for salt-treated seedlings resulted in significant gradual increases in the contents of GSH (Figure 2A) and AsA (Figure 2B), and in the ratios of GSH/GSSG (Figure 2E) and AsA/DHA (Figure 2F), while leading to significant gradual decreases in the contents of GSSG (Figure 2C) and DHA (Figure 2D). MT concentration of 150 µM (T4) was the best treatment as it exceeded (for GSH content and GSH/GSSG), equaled (for AsA and DHA contents), or did not reach the control values (for GSSG content and AsA/DHA). Generally, the salt application to the peanut seedlings for 6 h significantly exceeded the salt application for 15 days, with some fluctuations, for all tested antioxidative components (Figure 2). For the 150 µM of MT (T4) as the best treatment, the 15 days salt treatment significantly exceeded the 6 h salt treatment for all tested antioxidative tested parameters, except for GSSG content and AsA/DHA ratio (Figure 2).

**Figure 3:**
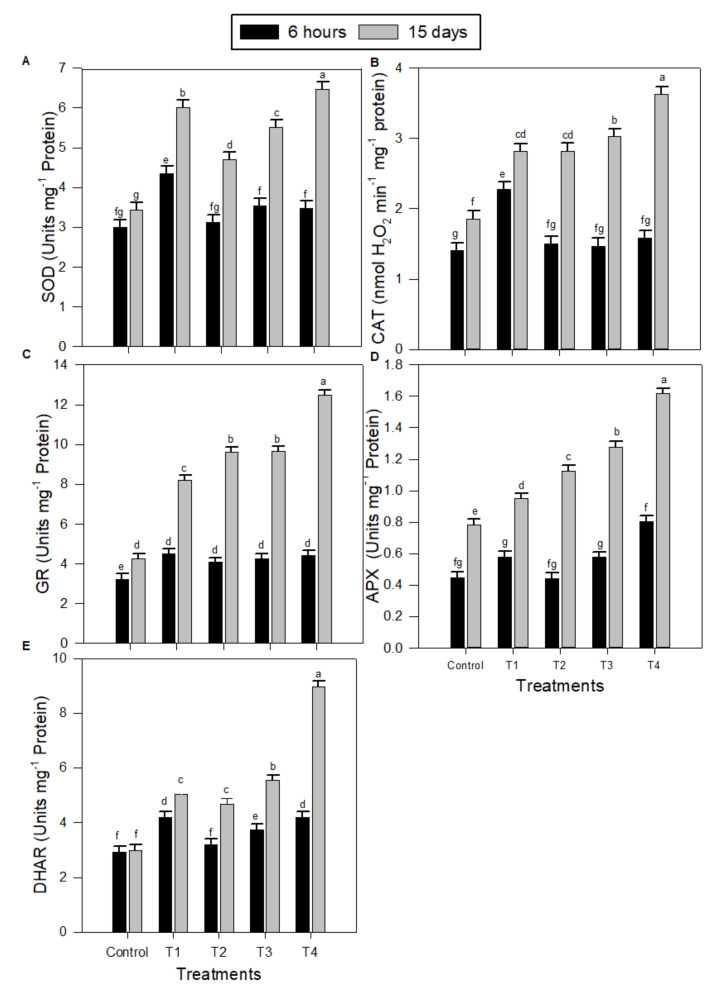
Effects of salinity stress combined with exogenous melatonin on the activities of antioxidant enzymes in peanut seedlings after 6 h and 15 days. (**A**) Superoxide dismutase (SOD), (**B**) catalase (CAT), (**C**) glutathione reductase (GR), (**D**) peroxidase (APX), and (**E**) dehydroascorbate reductase (DHAR). Data are means ± SD (*n* = 3) from three independent experiments. The bars labelled with different letters are significantly different. The significant difference marked by different letters was calculated using the Student’s *t*-test and further analyzed with the Fisher LSD test, at *p* ≤ 0.05. The legend is as follows: Control: 0 NaCl + 0 MT, T1: 150 mM NaCl + 0 MT, T2: 150 mM NaCl + 50 μM MT, T3: 150 mM NaCl + 100 μM MT, T4: 150 mM NaCl + 150 μM MT.

The activities of antioxidative enzymes superoxide dismutase (SOD) (Figure 3A), catalase (CAT) (Figure 3B), glutathione reductase (GR) (Figure 3C), peroxidase (APX) (Figure 3D), and dehydroascorbate reductase (DHAR) (Figure 3E) were significantly increased in the leaves of peanut seedlings as a result of salinity stress (T1) compared to control. However, the exogenous use of MT applied at 50–150 µM (T2, T3, and T4) for salt-treated seedlings resulted in significant gradual improvements in the activities of all tested enzymes. MT concentration of 150 µM (T4) was the best treatment. Generally, the impact of a MT application on peanut salt-stressed seedling after 15 days was significantly higher than the impact after 6 h.

### 2.3. Levels of Transcription of Gene-encoding Antioxidative Enzymes in Peanut Seedlings under Salinity Stress and Melatonin (MT) Treatments

**Figure 4:**
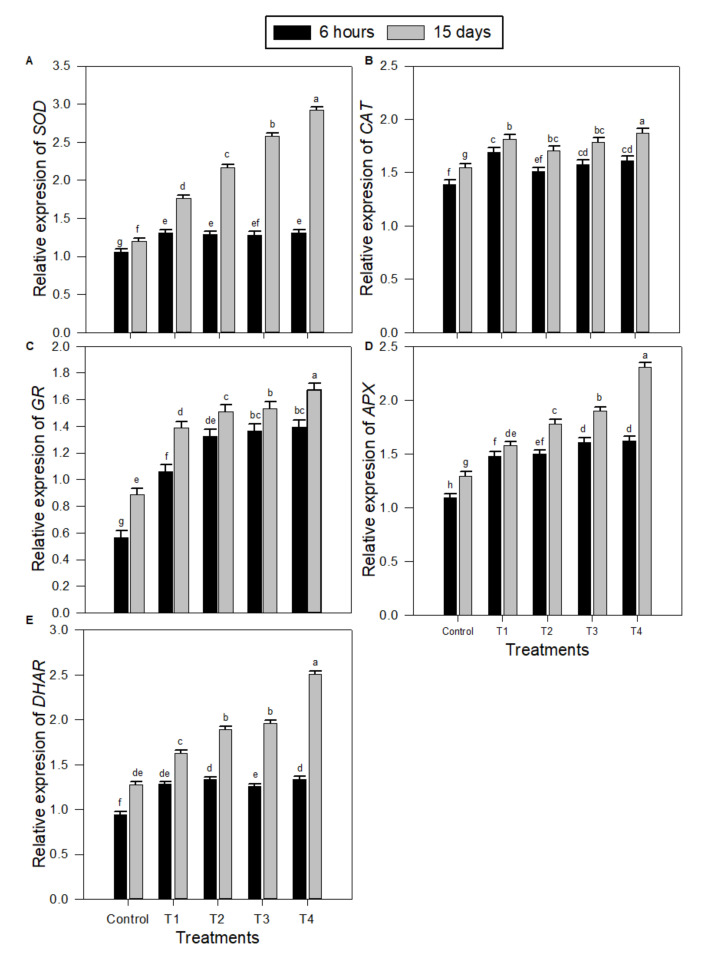
Transcript levels of antioxidant enzymes encoding genes in peanut seedlings under salinity stress combined with different exogenous melatonin treatments after 6 h and 15 days. (**A**) Superoxide dismutase (*SOD*), (**B**) catalase (*CAT*), (**C**) glutathione reductase (*GR*), (**D**) peroxidase (*APX*), and (**E**) dehydroascorbate reductase (*DHAR*). Transcript levels were quantified by qPCR and normalized against *actin* and *GAPDH* transcript levels. qPCR experiments were repeated three times with two technical replicates each. The bars labelled with different letters are significantly different. The significant difference marked by different letters was calculated using the Student’s *t*-test and further analyzed with the Fisher LSD test, at *p* ≤ 0.05. The legends are as follows: Control: 0 NaCl + 0 MT, T1: 150 mM NaCl + 0 MT, T2: 150 mM NaCl + 50 μM MT, T3: 150 mM NaCl + 100 μM MT, T4: 150 mM NaCl + 150 μM MT.

Quantitative RT-PCR (qRT-PCR) was used to compare transcription levels of different antioxidative enzyme-encoding genes in salt-stressed and unstressed peanut seedlings without or with the exogenous use of MT at different concentrations (Figure 4). The expressions of *SOD* (Figure 4A), *CAT* (Figure 4B), *GR* (Figure 4C), *APX* (Figure 4D), and *DHAR* (Figure 4E) encoding genes were highly expressed in peanut seedlings as a result of salinity stress treatment (T1) compared to control. However, the exogenous use of MT applied at 50–150 µM for salt-treated seedlings resulted in significant gradual increases in the expressions of SOD, CAT, GR, APX, and DHAR-encoding genes. MT concentration of 150 µM (T4) was, in general, the best treatment, as it exceeded the control values for the expressions of all antioxidative-encoding genes. Usually, the impact of MT application on peanut salt-stressed seedling after 15 days was significantly higher than the impact after 6 h for all tested antioxidative-encoding gene expressions under all MT treatments, especially T4.

### 2.4. Levels of Transcription of Melatonin (MT) Biosynthesis Genes in Peanut Seedlings under Salinity Stress and MT Treatments

**Figure 5:**
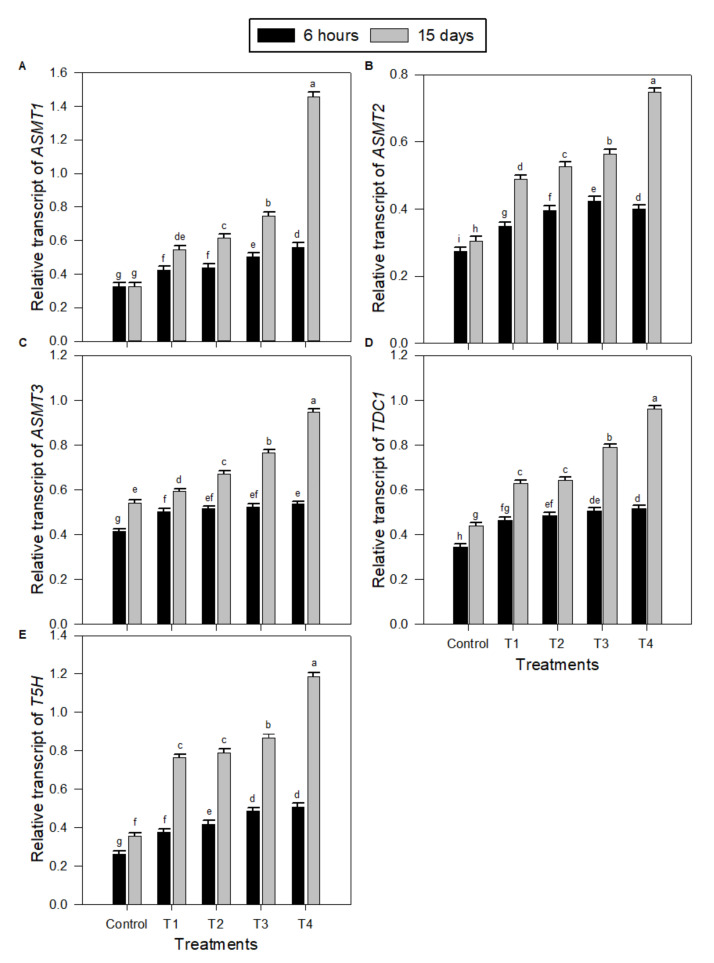
Transcript levels of melatonin biosynthesis encoding genes in peanut seedlings under salinity stress combined with different exogenous melatonin treatments after 6 h and 15 days. (**A**) *ASMT1*, (**B**) *ASMT2*, (**C**) *ASMT3*, (**D**) *TDC*, and (**E**) *T5H*. Transcript levels were quantified by qPCR and normalized against *actin* and *GAPDH* transcript levels. qPCR experiments were repeated three times with two technical replicates each. The bars labelled with different letters are significantly different. The significant difference marked by different letters was calculated using the Student’s *t*-test and further analyzed with the Fisher LSD test, at *p* ≤ 0.05. The legend is as follows: Control: 0 NaCl + 0 MT, T1: 150 mM NaCl + 0 MT, T2: 150 mM NaCl + 50 μM MT, T3: 150 mM NaCl + 100 μM MT, T4: 150 mM NaCl + 150 μM MT.

The relative expression of the MT biosynthesis genes *ASMT1* (Figure 5A), *ASMT2* (Figure 5B), *ASMT3* (Figure 5C), *TDC* (Figure 5D), and *T5H* (Figure 5E) were assessed in salt-stressed and unstressed peanut seedlings without or with exogenous use of 50, 100, or 150 µM MT. The expressions of *ASMT1, ASMT2, ASMT3*, *TDC*, and *T5H* genes were significantly elevated in peanut seedlings as a result of salinity stress treatment (T1) compared to control. Additionally, the exogenous use of MT applied at 50–150 µM (T2, T3, and T4) for salt-treated seedlings resulted in further increases in the expressions of all the MT biosynthesis genes. MT concentration of 150 µM (T4) was the best treatment, inducing the highest increases in the expressions level of all the MT biosynthesis genes. Furthermore, the impact of MT application on peanut salt-stressed seedling after 15 days was significantly higher than the impact after 6 h for all the MT biosynthesis genes: *ASMT1*, *ASMT2*, *ASMT3*, *TDC*, and *T5H* expressions under all MT treatment, especially T4 (Figure 5).

### 2.5. Nucleotide Identity Comparison and Phylogenetic Analyses

**Figure 6:**
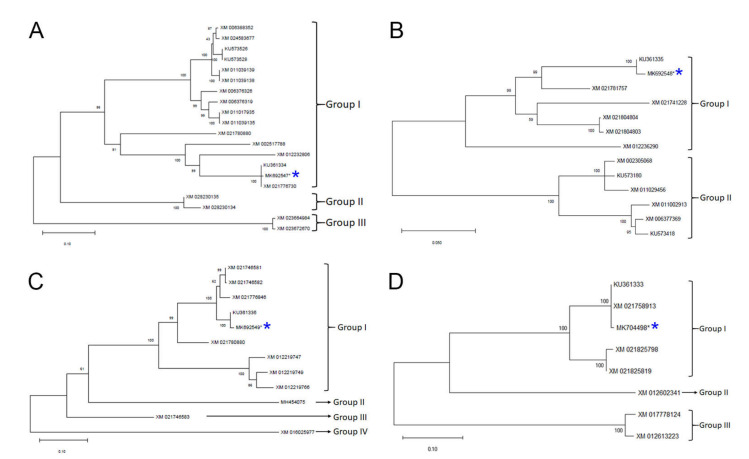
The rectangular form of a phylogenetic tree of a (**A**) *ASMT1*, (**B**) *ASMT2*, (**C**) *ASMT3*, and (**D**) *T5H* genes sequence produced by the maximum likelihood algorithm under the assumption of T92 (Tamura 3-parameter) substitution model coupled to a discrete gamma distribution (+G) and an assumed certain fraction of sites that are an evolutionarily invariable (+I) option of the MEGA-X version 10.0.5 software (Kumar et al., 2018). Three major groups were delineated. The numbers above the branches indicate the bootstrap confidence value. The scale bar shows the number of substitutions per nucleotide. The blue asterisk indicates the sequence amplified from *Arachis hypogaea*.

Nucleotide identity among twenty accession sequences ranged from 56.7% to 100.0% for *ASMT1*. Bioinformatic analysis indicated that the most divergent accessions from each other were (*Cucurbita pepo* subsp. Pepo; XM_023672670) and (*Jatropha curcas*; XM_012232806). Pairwise comparisons among the accessions of *Manihot esculenta* (XM_021776730 and KU361334) revealed that they were 100.0% identical as well as for the accessions of *Populus euphratica* (XM_011039138 and XM_011039139). The pairwise identity among the 20 accessions for the predicted amino acid comparisons was ranged from 3.4% to 100.0%. Consequently, the most distant accessions were XM_023672670 (*Cucurbita pepo* subsp. Pepo) and XM_021780880 (*Hevea brasiliensis*). In contrast, 100.0% identity was explored in the sequences of the accession couples mentioned above, i.e., *Manihot esculenta* (KU361334 with XM_021776730) and *Populus euphratica* (XM_011039138 with XM_011039139), respectively. Although the accession MK692547, the objective of this study, the most divergent from accession XM_023672670 (57.0% of identity), it was closer to the accessions KU361334 and XM_021776730 (99.7% of identity) at the nucleotide level. In contrast, it was most distant from the accession XM_006376326 (4.6% of identity), but closer to the accession XM_021780880 (24.9% of identity) at the predicted amino acids level. Evolutionary relationships among the 20 sequences of *ASMT1* were performed using the maximum likelihood (ML) approach and phylogenetically divided into three different groups. Group I encompassed 16 accessions. Remarkably, the accession MK692547 of *A. hypogaea* was closer to the accessions KU361334 and XM_021776730 of *Mahinot esculenta* than any other members of Group I (Figure 6A). On the other hand, Group II comprised of two accessions of *Camellia sinensis* (XM_028230134 and XM_028230135) and Group III comprised of two accessions of *Cucurbita pepo* subsp. Pepo (XM_023664984 and XM_023672670).

Nucleotide identity for *ASMT2* based on the comparison among 13 accessions was ranged from 74.3% to 99.6%. The two accessions of *Populus euphratica* (XM_011002913) and *A. hypogaea* (MK692548) were the most distant from one another while the two accessions of *Hevea brasiliensis* (XM_021804803 and XM_021804804) were closer to each other. Furthermore, the pairwise identity of 13 accessions for the predicted amino acid comparisons fluctuated from 63.2% to 99.4%. Accordingly, the accessions of *Populus tomentosa* (KU573418) and *A. hypogaea* (MK692548) were the most distant, while the accessions of *Hevea brasiliensis* (XM_021804803 and XM_021804804) were closest to each other. It is worth noting that although the accession of *A. hypogaea* (MK692548) was divergent the most from the accession of *Populus tomentosa* (KU573418), it was much closer to the accession XM_021804803 in terms of nucleotides and predicted amino acids and the intervals were as follows: 74.3–85.5% and 63.2–76.8%, respectively. Only two major phylogroups were outlined in the 13 *ASMT2* sequences. As shown in Figure 6B, whole sequences were posted unevenly into two clusters. Seven accessions were assigned to Group I whereas six accessions were assigned to Group II. The results revealed that the accession MK692548 of *A. hypogaea* was evolutionarily closely related to the accession KU361335 of *Manihot esculenta* and the accession XM_021781757 of *Hevea brasiliensis* (Figure 6B).

With respect to *ASMT3*, a pairwise nucleotides predicted amino acids comparison among 12 accessions showed that the highest divergence was noted between the accessions of *A. hypogaea* (MK692549) and *Ziziphus jujube* (XM_016025977), while the accession of *Manihot esculenta* (XM_021746582) was most similar to the accession of *Manihot esculenta* (XM_021746581). The corresponding intervals were as follows: 50.0–99.8% and 35.0–99.7%, respectively. It is worthwhile to explore that, at nucleotide and amino acid (predicted) level, the accession of *Manihot esculenta* (KU361336) was the closest to *A. hypogaea* (MK692549), but the accession of *Ziziphus jujube* (XM_016025977) was the farthest from it. The respective intervals were as follows: 50.0–99.3% and 39.0–98.2%, respectively. In comparison to *ASMT1* and *ASMT2*, concluded phylogeny of twelve sequences of *ASMT3* exhibited that they are more heterogeneous than the former two variants as they were represented into four different groups. Group I contained nine accessions, while Group II, Group III, and Group IV were represented by a single accession accessions of *Tabernanthe iboga*, *Manihot esculenta*, and *Ziziphus jujube* (MH454075, XM_021746583, and XM_016025977, respectively) (Figure 6C). Consequently, the accession MK692549 of *A. hypogaea* was phylogenetically closely related to the accession KU361336 of *Manihot esculenta*, and to three accessions of *Manihot esculenta* (XM_021776846, XM_021746582, and XM_021746581).

With regard to T5H, the pairwise identity for the nucleotide identity and the predicted amino acid comparisons among eight accessions delineated two pairs of accessions of *Gossypium arboreum* and *Hevea brasiliensis* (XM_017778124 and XM_021825798, respectively), and *Manihot esculenta* (XM_021758913, KU361333) with the respective intervals: 41.3–100.0% and 32.7–100.0%, respectively. Concerning the accession of *A. hypogaea* (MK704498) in this study, it was shown that it was closest to the accession of *Manihot esculenta* (XM_021758913) but most distant from the accession of *Gossypium arboreum* (XM_017778124) at nucleotide as well as predicted amino acid levels with the following intervals: 45.4–99.5% and 38.1–98.8%, respectively. Relating to TDC, only two sequences were available in the databases for *Manihot esculenta* (XM_021773503 and KU361330). These two sequences were 100.0% identical both on the level of nucleotides and predicted amino acids. The compared accession of *A. hypogaea* (MK692550) to the former two pointed out that it differed a little from both sequences, i.e., 99.5% and 98.6% of nucleic acids and predicted amino acids identity, respectively. Phylogeny of eight accession sequences of T5H result in three separated groups (Figure 6D). Group I assembled five accessions, Group II was comprised by a single accession, and Group III included two accessions. The tree topology clearly showed that, even with this protein, the accession MK704498 of *A. hypogaea* was revealed to be phylogenetically closer to the accessions of *Manihot esculenta* (KU361333 and XM_021758913) (Figure 6D). Lastly, it is noteworthy that no tree reconstruction was possible for the TDC protein since three highly similar sequences (the two *Manihot esculenta* accessions XM_021773503 and KU361330, and the *A. hypogaea* accession MK692550) available in databases could not provide reliable results.

### 2.6. Maximum Likelihood Estimation of Substitution Matrix and Transition/Transversion Bias

Substitution patterns play an important role in the evolutionary process that may influence the activity, functions, and durability of a protein. In order to determine the substitution patterns and rates in the five melatonin biosynthesis genes in this study, a substitution matrix was estimated for each type of protein. Thus, for *ASMT1*, it was shown that rates of different transitional substitutions ranged from 13.5 to 17.2, whereas rates of transversional substitutions varied from 4.2 to 5.6 (Table 1). Concerning *ASMT2*, rates of transitional substitutions oscillated between 12.0 and 17.2, while rates on transversional substitutions ranged from 4.1 and 6.5. With regard to *ASMT3*, although rates of transitional substitutions varied from 10.9 to 17.2, those of transversional substitutions oscillated between 4.1 and 6.4. As for the previous genes, an estimation of the substitution matrix in *T5H* revealed the following intervals: transitional substitutions (11.0–17.2) and transversional substitutions (4.1–9.4). For the *TDC*, the unique rate had the value 8.3 across the range (Table 1). The estimated transition/transversion biases were as follows: *ASMT1* (1.5), *ASMT2* (1.3), *ASMT3* (1.3), *T5H* (1.2), and *TDC* (0.5) (Table S1). For estimating ML values, a tree topology was computed for each type of protein. These analyses involved 20 (*ASMT1*), 13 (*ASMT2*), 12 (*ASMT3*), 8 (*T5H*), and 3 (*TDC*) nucleotide sequences. Codon positions involved were 1st + 2nd + 3rd + noncoding. There were 1188 (*ASMT1*), 1098 (*ASMT2*), 1119 (*ASMT3*), 1611 (*T5H*), and 1491 (*TDC*) positions in the final dataset.

## 3. Discussion

Recently, melatonin has emerged as a possible plant growth regulator, exogenously applied melatonin can enhance defense responses to different abiotic and biotic stress of plants by regulating both the enzymatic and non-enzymatic antioxidant defense systems [24,25,26,27,28,29]. Nevertheless, there is inadequate knowledge about the defense mechanism of melatonin to salinity stress. In the present study, the results indicated that an application of exogenous melatonin showed a positive protecting role of melatonin in peanut seedlings against salinity stress. The results revealed that salinity stress-induced increase in MDA was consistent with the accumulation of H_2_O_2_ (Figure 1A,B), respectively. Remarkably, in the existence of exogenous melatonin, the accumulation level of H_2_O_2_ and MDA were significantly reduced in peanut seedlings under salinity stress (T2, T3, and T4). Therefore, it is possible that melatonin at a high concentration (150 µM, T4) could act as an antioxidant and is engaged in the ROS-scavenging. The results in the current study suggest that the protective effect of exogenous melatonin against oxidative damage in plants appears to rely on the melatonin concentration. Previous studies indicated that exogenous melatonin plays significant roles in improving environmental stress-induced oxidative stress by scavenging ROS in plants [16,22,24,26,28,30,31,32]. Furthermore, melatonin may play an essential role as a scavenger of ROS and reactive nitrogen species (RNS), lipid peroxides, and toxic chemicals, to maintain redox homeostasis and protect the cell membrane against damage [25,33,34]. In contrast, previous studies showed strong evidence that melatonin is incapable of directly scavenge O_2_^•−^ and H_2_O_2_ [35,36], and, therefore, the regulation of redox homeostasis by melatonin results from its capability to provoke antioxidant systems comprising enzymatic and non-enzymatic antioxidants [24].

**Figure 7:**
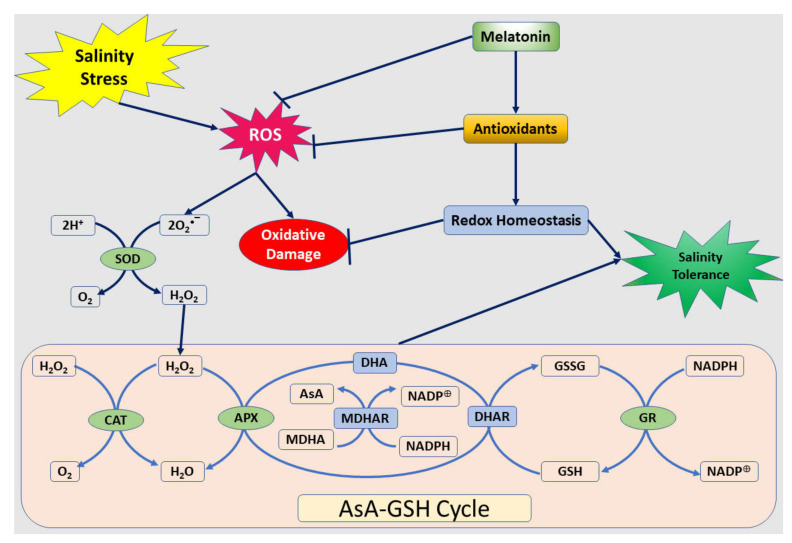
Schematic presentation of the ascorbate–glutathione cycle and a model depicting the melatonin-induced alleviation of NaCl-caused oxidative stress in peanut plants. Abbreviations: ROS, reactive oxygen species; SOD, superoxide dismutase; CAT, catalase; APX, ascorbate peroxidase; AsA, ascorbate; MDHA(R), monodehydroascorbate (reductase); NADPH, nicotinamide adenine dinucleotide phosphate, DHA, dehydroascorbate; DHAR, dehydroascorbate reductase; GSH, reduced glutathione; GSSG, oxidized glutathione; and GR, glutathione reductase.

SOD is considered to be the front line of defense in plant cells against oxidative damage, although H_2_O_2_ is also scavenged by an AsA and/or a GSH regenerating cycle and CAT [37]. It has been previously found that exogenous melatonin treatments enhanced AsA and GSH levels in plants and up-regulated the activities of antioxidant enzymes such as SODs, CATs, and peroxidases [18,38]. Similarly, the results in the current study show that AsA and GSH levels, the activities of DHAR, APX, and GR, and the redox status involved in the AsA–GSH cycle markedly increased with the exogenous melatonin treatment, particularly at higher concentration (150 µM) under salinity stress (Figure 2 and Figure 3). The AsA–GSH cycle in plants is well characterized as an antioxidant system against oxidative damage [32]. Stimulus of the AsA–GSH cycle is proposed to be a vital mechanism of salinity tolerance [39]. The AsA–GSH cycle consists of a network of reactions involving APX and enzymes that thereafter serve to regenerate ascorbate. Therefore, these findings indicate that an exogenous melatonin treatment could enhance plant cellular redox homeostasis by triggering the whole antioxidant system to protect cells injury caused by oxidative stress due to salinity stress (Figure 7).

The results of qRT-PCR revealed that salinity stress imposed on peanut seedlings, together with exogenous melatonin, up-regulated gene expression of *SOD, CAT, GR, APX*, and *DAHR* genes (Figure 4). Rapid detoxification of O_2_^•−^ requires high SOD activity, for example, to diminish lipid peroxidation and peroxinitrate configuration if nitric oxide is produced concurrently [40], which is in well harmony with the present study where melatonin stimulated activities of SOD under salinity stress. Additionally, generated H_2_O_2_ must be detoxified; this is accelerated by enhanced activities of CAT, APX.

In plants, tryptophan is the precursor of the melatonin biosynthesis via activities of four sequential enzymes (TDC, T5H, SNAT, and ASMT). In rice, melatonin biosynthesis pathways were found to be induced by multiple stresses via the changes in the transcript levels of genes involved in this process [41,42]. Therefore, the current study was carried out to investigate whether the same genes in peanut seedlings were up-regulated under salinity stress conditions and different concentrations of exogenous melatonin. In the present study, the expression level of the four melatonin biosynthesis genes *ASMT*, *TDC*, *SNAT*, and *T5H* was significantly increased in the peanut seedlings treated with NaCl compared with non-treated plants (Figure 5). Li et al. [13] found that the melatonin biosynthesis genes (*ASMT*, *TDC*, *SNAT*, and *T5H*) were up-regulated under drought conditions in two *Malus* species. Furthermore, salinity stress stimulated a significant increase in serotonin and melatonin contents in sunflower seedlings [19]. TDC is the first enzyme in the melatonin biosynthetic pathway which catalyzes the conversion of tryptophan into tryptamine [43,44] and is localized in the cytoplasm [45], whereas T5H is the second enzyme in the pathway, which is localized in the endoplasmic reticulum (ER) [46]. The last enzyme in the melatonin biosynthetic pathway is ASMT, which is localized in the cytoplasm, while SNAT is also involved in the melatonin biosynthesis pathway and resides in chloroplasts, thus, the melatonin biosynthesis in plants takes place in three different cellular compartments (the cytoplasm, the ER, and chloroplasts) [47].

Regarding the four different genes involved in melatonin biosynthesis in plants, the *T5H* [46] and *SNAT* [12] occur as single isoform, whereas *TDC* [43,48] and *ASMT* [11] belong to small gene families each with at least three isoforms. Similarly, the sequence analysis isolated from *Arachis hypogaea* in the present study resulted in three isoforms of *ASMT*. Therefore, the presence of gene families for the melatonin biosynthetic pathway in plants indicates the complex regulation and functional distinction in specific gene isoforms. Nevertheless, no functional roles for these gene families in melatonin biosynthesis have been characterized to date for either *ASMT* or *TDC*. Little is known about the relationships between plant melatonin biosynthetic genes. In the present study, a pairwise nucleotide and predicted amino acid analysis among 56 sequences (20 *ASMT1*, 13 *ASMT2*, 12 *AMST3*, 8 *T5H*, and 3 *TDC*) and inferred phylogenies led to the conclusion that sequences were very heterogeneous and that *A. hypogaea* accessions were primarily closest to those of cassava (*Manihot esculenta*) [49]. This suggests that the *ASMT* and *T5H* are orthologs of cassava *ASMT*. Further research on evolutionary aspects could shed more light on the genetic relationship between these two plant species. In conclusion, the phylogenetic and sequence analysis of the current study showed that the three *ASMT* isoforms isolated from *A. hypogaea* were reported to be encoded by different genes. A similar report was obtained for rice [42]. Accordingly, further studies on the regulation and function of *TDC*, *T5H*, *SANT*, and *ASMT* melatonin biosynthetic genes will help to understand the role of melatonin in plants, especially under different stress responses. In addition, a further study should consider the assessment of the metabolites involved in melatonin production.

## 4. Materials and Methods

### 4.1. Plant Material and Treatments

Peanut (*Arachis hypogaea* L.) seeds (cv. NC-9) were obtained from Horticultural Sciences Department, College of Agriculture, Zagazig University, Egypt, and surface sterilized in a 1% (*v*/*v*) sodium hypochlorite solution for 10 min, then the seeds were rinsed several times with sterile water. Peanut seeds germination was done by placing peanut seeds in Petri dishes (Fisher Scientific, Pittsburgh, PA, USA) covered with moist filter paper (Whatman No. 1, GE Healthcare Bio-Sciences, Pittsburgh, PA, USA). Petri dishes were placed in the dark at 25 °C. Each healthy germinated seed was sown in a plastic container (7 × 8.5 × 10 cm) filled with a mixture of autoclaved cleaned sand and commercial peat-based compost fertilizer (2:1, *v*/*v*). The seedlings were pre-cultured in a greenhouse under natural light located at the College of Agriculture, Zagazig University, Zagazig, Egypt (30°43′54″ N; 30°33′01″ E). In this location, the greenhouse conditions were 27–35 °C/16–20 °C (day/night) and 60–70% relative humidity. Each treatment included three pots with a single plant in each pot. All seedlings were regularly watered with the same amount of ½ strength Hoagland’s solution [50]. The melatonin (MT) (Sigma-Aldrich, Seelze, Germany) solutions were prepared by dissolving the solute in ethanol. The resultant was then diluted with deionized water to reach the dilution rate (ethanol/water (*v*/*v*) = 1/10,000). At the three-leaf stage, the germinated seedlings were divided uniformly into five groups (Control, Treatments 1, 2, 3, and 4, designated T1, T2, T3, and T4, respectively). Control, T1, T2, T3, and T4 received 100 mL per seedling of distilled water, 150 mM NaCl, 150 mM NaCl + 50 µM MT, 150 mM NaCl + 100 µM MT, 150 mM NaCl + 150 µM MT, respectively, onto the roots. These treatments were provided at two-day intervals along with the nutritive solution until the termination of the experiment (after 15 days). To avoid osmotic shock, the seedlings were exposed to an initial concentration of 50 mM NaCl that was increased gradually to 150 mM (the needed concentration) through three irrigations. The experimental treatments were organized in a completely randomized design with three replications (each replication consisted of 4 containers) and the experiment was repeated over time for a set of experiments. Leaves samples of fresh shoots were harvested after 6 h and 15 days of MT and NaCl treatments. All samples were washed with deionized water in order to eliminate MT and salt residues then directly placed in liquid N_2_ and stored at −80 °C until starting the biochemical assays.

### 4.2. Determination of Malondialdehyde (MDA) Contents

MDA contents were determined in peanut seedling leaves after 6 h and 15 days of NaCl and MT treatments. Frozen samples of peanut leaves (300 mg) were homogenized in 5% trichloroacetic acid (TCA) on ice. After that, the mixture was centrifuged at 12,000× *g* at 4 °C for 10 min. The pellet was extracted twice with the same solvent. A total of 0.5 mL of supernatant was added to 1.5 mL of 5% TCA containing 0.67% thiobarbituric acid (TBA) and then heated to 95 °C in a water bath for 25 min. Next, the samples were cooled down to room temperature and then centrifuged at 1200× *g* for 10 min. The samples’ absorption was measured at 532 nm using UV spectrometer with distilled water as a blank and normalized by reading at 600 nm for the non-specific absorption [51]. The MDA content was calculated as μg MDA g^−1^ fresh weight (FW).

### 4.3. Determination of Hydrogen Peroxide (H_2_O_2_) Content

Hydrogen peroxide content was extracted with 5% (w/v) TCA and measured according to the methods of Willekens et al. [52].

### 4.4. Determination of Proline Content

Proline contents were determined in peanut seedling leaves after 6 h and 15 days of NaCl and MT treatments, respectively, as described by Vicente et al. [53]. Proline contents in the plant samples were expressed as μmol proline per gram of FW.

### 4.5. Determination of GSH, GSSG, DHA, and AsA Contents

GSH and GSSG contents were assayed by an enzymatic recycling method as described by Rao et al. [54]. The assay for the dehydroascorbic acid (DHA) and ascorbic acid (AsA) content was done as described by Law et al. [55]. To obtain DHA concentration, the AsA concentration was subtracted from the total concentration.

### 4.6. Determination of Antioxidant Enzyme Activities

Fresh peanut leaves (500 mg) were ground to fine powder in liquid N_2_ and extracted with chilled extraction phosphate buffer (pH 7.0). The homogenate was centrifuged at 12,000× *g* for 30 min at 4 °C. The supernatant was used to measure total soluble protein and various antioxidant enzyme activities. Total soluble protein content was measured by using the Bradford’s method [56]. SOD activity was determined by the methods of Zhang et al. [14]. One unit of SOD activity was defined as the amount of enzyme causing 50% inhibition of photochemical reduction of NBT (nitroblue tetrazolium). CAT activity was measured according to the method of Aebi [57]. Activity of CAT was calculated as reduced H_2_O_2_ mg^−1^ protein min^−1^. APX was assayed following the procedure of Dzung et al. [58]. The activity of GR was assayed following the method of Mandhania et al. [59]. The activity of DHAR was estimated according to the method of Mishra et al. [60]. DHAR activity was defined as μmol DHA reduced mg^−1^ protein min^−1^.

### 4.7. Total RNA Isolation and Quantitative Real-time PCR (qRT-PCR) Amplification

Total RNA was extracted from 200 mg peanut leaves using the RNase Plant Mini Kit (Qiagen, Valencia, CA, USA) and used for cDNA synthesis using a QuantiTect Reverse Transcription Kit (Qiagen). The transcription levels of APX, SOD, CAT, DHAR, GR, 1-Cys-Prx, PrxQ, TDC, T5H, ASMT1, ASMT2, and ASMT3 were analyzed by qRT-PCR using an iCycler Thermal Cycler (Bio-Rad Laboratories, Inc., Hercules, CA, USA) with a SYBR Green Master Mix (Bio-Rad) according to the manufacturer’s instructions. The primers used for the amplification of the previous genes are listed in Table S2. The transcription levels were normalized against the peanut actin and GAPDH genes (an internal control), which were constantly expressed under all experimental conditions. For relative quantification, change in gene expression was calculated by the ^2−∆∆^CT method as described by Livak and Schmittgen [61].

### 4.8. Cloning and Sequencing of Melatonin Biosynthesis Genes

The PCR amplicons of the melatonin biosynthesis genes (*ASMT1*, *ASMT2*, *ASMT3*, *TDC*, and *T5H*) derived from reverse transcription RT-PCRs that were cloned in the pGEM-T Easy vector system (Promega Corporation, Madison, WI, USA). The recombinant plasmids containing the inserts were purified using the Pure Yield Plasmid Miniprep system (Promega Corporation). The clones were selected for sequencing using an automated sequencer (ABI PRISM 3100 Genetic Analyzer: Applied Biosystems, Foster City, CA, USA) by using universal M13 sequencing primers and internal primers specific for each of the melatonin biosynthesis genes. One clone per amplicon was sequenced and used for alignment and phylogenetic analysis.

### 4.9. Sequence Submission to GenBank

The sequences of five genes encoding protein, which amplified from *A. hypogaea*, were deposited in the GenBank database at www.ncbi.nlm.nih.gov under the following accession numbers: *ASMT1* (MK692547), *ASMT2* (MK692548), *ASMT3* (MK692549), *T5H* (MK704498), and *TDC* (MK692550).

### 4.10. Sequences Alignment and Construction of Phylogenetic Trees

GenBank data searches for homologies to sequences of three variants of ASMT (*ASMT1*, *ASMT2*, and *ASMT3*), *T5H*, and *TDC* were accomplished using the FASTA according to Pearson and Lipman [62] and WU-BLAST 2, based on the Basic Local Alignment Search Tool (BLAST) algorithm [63,64] programs. In addition, the investigated sequences included other available accessions retrieved from the GenBank (Table S3).

The CLUSTAL X 2.1 [65] software was used to align the nucleotide sequences with the default settings. The phylogenetic relations among the sequences were defined using the maximum likelihood (ML) algorithm included in the MEGA-X version 10.0.5 program [66]. Based on the assessment of best fit substitution model implemented in MEGA-X, the ML tree was rebuilt under the assumption of various substitution models. The model used was Tamura 3-parameter [67] coupled or not coupled to a separate Gamma distribution (G) with 5 rate groups and assuming or not assuming that a certain fraction of sites is evolutionarily invariable (I). Thus, the best fit models were as follows: *ASMT1* (T92 + G + I), *ASMT2* (T92 + I), *ASMT3* (T92 + G), and *T5H* (T92 + G). However, regarding *TDC*, the best fitting model was JC [68]. The selection of the substitution model was performed based on Bayesian Information Criterion (BIC) values acquired. Therefore, the lowest BIC value among 24 models tested, the best description of the substitution pattern. Additional parameters resulting from the analysis are mentioned in Table S1.

### 4.11. Statistical Analyses

The experiment was a completely randomized design with three replicates. All data were subjected to analysis of variance (ANOVA) [69] using the MSTAT-C statistical package [70]. Mean separation was performed according to Fisher’s Protected LSD test at *p* < 0.05.

## Figures and Tables

**Table 1:** Description of the maximum likelihood estimate of the pattern of nucleotide substitution in the five melatonin biosynthesis genes. Each entry is the probability of substitution from one base (row) to another base (column). Rates of different transitional substitutions are shown in bold and the rest for transversional substitutions.

Gene	Nucleotide Substitution Matrix				
		A	T	C	G
*ASMT1*	A	-	5.31	4.17	**13.63**
	T	5.55	-	**13.47**	4.58
	C	5.55	**17.17**	-	4.58
	G	**16.51**	5.31	4.17	-
*ASMT2*	A	-	5.88	4.09	**12.04**
	T	6.50	-	**11.96**	4.86
	C	6.50	**17.22**	-	4.86
	G	**16.12**	5.88	4.09	-
*ASMT3*	A	-	6.40	4.06	**12.48**
	T	6.36	-	**10.91**	5.08
	C	6.36	**17.80**	-	5.08
	G	**15.63**	6.40	4.06	-
*T5H*	A	-	6.39	4.09	**12.32**
	T	6.40	-	**10.98**	5.02
	C	6.40	**17.18**	-	5.02
	G	**15.72**	6.39	4.09	-
*TDC*	A	-	8.33	8.33	**8.33**
	T	8.33	-	**8.33**	8.33
	C	8.33	**8.33**	-	8.33
	G	**8.33**	8.33	8.33	-

A = adenine, T = thymine, C = cytosine, G = guanine, *ASMT* = N-acetylserotonin methyltransferase, *T5H* = tryptamine 5-hydroxylase, and *TDC* = tryptophan decarboxylase.

## Supplementary Materials

The following are available online at https://www.mdpi.com/2223-7747/9/7/854/s1, Table S1: For each protein-coding gene, a model with a BIC score (Bayesian Information Criterion) are considered to describe the substitution pattern the best. For each model, non-uniformity of evolutionary rates among sites is modeled by using a discrete Gamma distribution (+G) with 5 rate categories and by assuming that a certain fraction of sites is evolutionarily invariable (+I). Assumed or estimated values of transition/transversion bias (R) for each model as well as nucleotide frequencies (f) and rates of base substitutions (r) for each nucleotide pair are shown. The analyses were conducted in MEGA X. Table S2: Primers sequences for semi-quantitative and quantitative RT-PCR of the salt-related genes in Peanut (*Arachis hypogaea* L. cv. NC-9) seedlings, Table S3: Description of 5 protein-coding genes extracted from different plant hosts and their corresponding accession numbers. The asterisk indicates the sequence amplified from *Arachis hypogaea* (this study).
