# Supplementary material for: Melatonin Regulatory Mechanisms and Phylogenetic Analyses of Melatonin Biosynthesis Related Genes Extracted from Peanut under Salinity Stress
Source: Plants (Basel). 2020 Jul 6;9(7):854. doi: 10.3390/plants9070854 (PMC7411912; doi:10.3390/plants9070854)
Supplement: Supplementary file 1 [file plants-09-00854-s001.pdf]

Melatonin regulatory mechanisms and phylogenetic analyses implying new sequences of melatonin biosynthesis related genes extracted from peanut under salinity-induced stress

Abdelaleim Ismail ElSayed[1], Moncef Boulila[2], Mohammed Suhail Rafudeen[3], Azza H. Mohamed[4,5], Sonali Sengupta[6], Mostafa Rady[7], Ahmad A. Omar[1,5]*

Supplementary Data

Supplementary Tables

Table S1. For each protein-coding gene, a model with a BIC score (Bayesian Information Criterion) are considered to describe the substitution pattern the best. For each model, non-uniformity of evolutionary rates among sites is modeled by using a discrete Gamma distribution (+G) with 5 rate categories and by assuming that a certain fraction of sites are evolutionarily invariable (+I). Assumed or estimated values of transition/transversion bias (R) for each model as well as nucleotide frequencies (f) and rates of base substitutions (r) for each nucleotide pair, are shown. The analyses were conducted in MEGA X

Type of protein	Model	BIC	+I	+G	R	f(A)	f(T)	f(C)	f(G)	r(AT)	r(AC)	r(AG)	r(TA)	r(TC)	r(TG)	r(CA)	r(CT)	r(CG)	r(GA)	r(GT)	r(GC)
ASMT1	T92+G+I	16059	0.18	3.29	1.51	0.277	0.277	0.223	0.223	0.055	0.044	0.135	0.055	0.135	0.044	0.055	0.168	0.044	0.168	0.055	0.044
ASMT2	T92+I	9609	0.37	-	1.32	0.290	0.290	0.210	0.210	0.062	0.045	0.120	0.062	0.120	0.045	0.062	0.167	0.045	0.167	0.062	0.045
ASMT3	T92+G	13984	-	1.72	1.25	0.291	0.291	0.209	0.209	0.064	0.046	0.117	0.064	0.117	0.046	0.064	0.164	0.046	0.164	0.064	0.046
T5H	T92+G	12551	-	1.23	1.24	0.292	0.292	0.208	0.208	0.064	0.046	0.117	0.064	0.117	0.046	0.064	0.164	0.046	0.164	0.064	0.046
TDC	JC	4218	-	-	0.50	0.250	0.250	0.250	0.250	0.083	0.083	0.083	0.083	0.083	0.083	0.083	0.083	0.083	0.083	0.083	0.083

Table S2: Primers sequences for semi quantitative and quantitative RT-PCR of the salt-related genes in Peanut (*Arachis hypogaea* L. cv. NC-9) seedlings

Gene name	Reference Sequence	Direction	5′ - 3′ primer sequence	T_A
Actin	XM_002448995.1	Forward	CGGTGCCAATCTATGAAGGT	58 °C
		Reverse	GCGAGCTTTTCCTTGATGTC	
GAPDH	M64116.1	Forward	TTTTCGGCATCAGGAACCCT	58 °C
		Reverse	AGCGTCTTTGCTGGGTTCAG	
DHAR	AY074784	Forward	TCAAGAACGTGACAAAGGTGG	58 °C
		Reverse	TAACGGTGGTGATGGCAAAT	
SOD	MG893090.1	Forward	TTCGCCATGCTGGTGATCTT	58 °C
		Reverse	CATGGACAACTACGGCCCTT	
CAT	GU984379	Forward	GGCTGCTTGAAGTTGTTCTCCT	58 °C
		Reverse	CTGCTAGTACCTCCTGATCCGTT	
APX	KU747079.1	Forward	TGGCCTGCTCTTCCTCTAGT	58 °C
		Reverse	CATGCCACGCTAATCGAAGC	
GR	KX828561.1	Forward	CAACGCGCTTTGGTAACTCC	58 °C
		Reverse	GGGCCCTAATGAAGTGGAGG	
1-Cys-Prx	AY304482	Forward	AATGGACCAAGGACATCGAG	58 °C
		Reverse	GACGGGTACAGGAAGCTCAG	
PrxQ	AY789643	Forward	ACTTCACGCTCAAGGACCAG	58 °C
		Reverse	CCGCCTTCTTGTACTTCTCG	
ASMT1	XM_021776730.1	Forward	GTTCAGCGATGGAGAGAGCA	58 °C
		Reverse	GACCAGCACCATCATGTCCA	
ASMT2	XM_021743110.1	Forward	CATTCCGGAGTTGGCTTCTG	58 °C
		Reverse	GTCCATGAAGAGCCTTGCCC	
ASMT3	XM_021778135.1	Forward	TTGGTCAGCGGAGAAACCATA	58 °C
		Reverse	CGGTCTGCTGCTCATTTCCA	
TDC	XM_021773503.1	Forward	AATTGGCTTGCTTCACCTGC	58 °C
		Reverse	TCGGATTTCACGAACCCCTC	
T5H	KU361333.1	Forward	CTCTTGCAATTCGGGCACAA	58 °C
		Reverse	TGGACTGTTGTCTTGGGAGG	

Table S3. Description of 5 protein-coding genes extracted from different plant hosts and their corresponding accession numbers. The asterisk indicates the sequence amplified from *Arachis hypogaea* (this study)

Gene	Protein	Plant host	Accession Numbers
ASMT1	trans-resveratrol di-O-methyltransferase-like	*Manihot esculenta*	XM_021776730
		Manihot esculenta	KU361334
		Ricinus communis	XM_002517788
		Jatropha curcas	XM_012232806
		Hevea brasiliensis	XM_021780880
		Populus trichocarpa	XM_006376319
		Populus euphratica	XM_011017935
		Populus euphratica	XM_011039135
		Populus trichocarpa	XM_006376326
		Populus euphratica	XM_011039139
		Populus euphratica	XM_011039138
		Populus tomentosa	KU573528
		Populus tomentosa	KU573526
		Populus trichocarpa	XM_006388352
		Populus trichocarpa	XM_024583677
		Camellia sinensis	XM_028230135
		Camellia sinensis	XM_028230134
		Cucurbita pepo	XM_023672670
		Cucurbita pepo	XM_023664984
		Arachis hypogaea	MK692547*
ASMT2	trans-resveratrol di-O-methyltransferase-like	*Manihot esculenta*	KU361335
		Hevea brasiliensis	XM_021781757
		Hevea brasiliensis	XM_021804804
		Hevea brasiliensis	XM_021804803
		Manihot esculenta	XM_021741228
		Jatropha curcas	XM_012236290
		Populus trichocarpa	XM_002305068
		Populus trichocarpa	XM_006377369
		Populus tomentosa	KU573180
		Populus euphratica	XM_011029456
		Populus euphratica	XM_011002913
		Populus tomentosa	KU573418
		Arachis hypogaea	MK692548*
ASMT3	trans-resveratrol di-O-methyltransferase-like	*Manihot esculenta*	KU361336
		Manihot esculenta	XM_021746582
		Manihot esculenta	XM_021746581
		Manihot esculenta	XM_021776846
		Hevea brasiliensis	XM_021780880
		Jatropha curcas	XM_012219747

		Jatropha curcas	XM_012219749
		Jatropha curcas	XM_012219766
		Ziziphus jujuba	XM_016025977
		Manihot esculenta	XM_021746583
		Tabernanthe iboganoribogaine	MH454075
		Arachis hypogaea	MK692549*
T5H	tryptamine 5-hydroxylase	*Manihot esculenta*	XM_021758913
		Manihot esculenta	KU361333
		Hevea brasiliensis	XM_021825819
		Hevea brasiliensis	XM_021825798
		Gossypium raimondii	XM_012602341
		Gossypium raimondii	XM_012613223
		Gossypium arboreum	XM_017778124
		Arachis hypogaea	MK704498*
